# Serum tenascin-C discriminates patients with active SLE from inactive patients and healthy controls and predicts the need to escalate immunosuppressive therapy: a cohort study

**DOI:** 10.1186/s13075-015-0862-4

**Published:** 2015-11-25

**Authors:** Jakub Závada, Michal Uher, Radka Svobodová, Marta Olejárová, Markéta Hušáková, Hana Ciferská, Hana Hulejová, Michal Tomčík, Ladislav Šenolt, Jiří Vencovský

**Affiliations:** Institute of Rheumatology, Prague, and Department of Rheumatology, First Faculty of Medicine, Charles University in Prague, Na Slupi 4, Praha 2, 12850 Prague, Czech Republic; Institute of Biostatistics and Analyses, Masaryk University, Brno, Czech Republic

**Keywords:** Tenascin-C, Biomarker, SLE, Disease activity, Flare

## Abstract

**Introduction:**

The aim of this study was to examine whether circulating levels of the proinflammatory glycoprotein tenascin-C (TNC) are useful as an activity-specific or predictive biomarker in systemic lupus erythematosus (SLE).

**Methods:**

Serum TNC levels were determined by enzyme-linked immunosorbent assay at inception visit in a prospective cohort of 59 SLE patients, and in 65 healthy controls (HC). SLE patients were followed for a mean of 11 months, disease activity was assessed using the Systemic Lupus Erythematosus Disease Activity Index 2000 (SLEDAI-2 K) and British Isles Lupus Assessment Group disease activity index (BILAG-2004), clinical and laboratory data were recorded every 3–6 months, and changes in glucocorticoids (GC) and immunosuppressants (IS) were recorded serially. We examined cross-sectionally the relationships between serum concentrations of TNC and SLE status, SLEDAI-2 K scores, strata of disease activity, and levels of conventional biomarkers [anti–double-stranded DNA (dsDNA), anti-nucleosome antibodies, C3 and C4]. We also explored the utility of TNC levels for predicting disease flares, defined as (i) new/increased GC, (ii) new/increased GC or IS, and (iii) increase in SLEDAI by ≥3 or (iv) BILAG A or B flare.

**Results:**

There was no significant difference in the mean levels of TNC between the SLE patients and HC. However, in SLE patients with active disease (SLEDAI ≥6), the TNC levels were significantly higher than in the HC (*p* = 0.004) or in patients with no/low disease activity (*p* = 0.004). In SLE patients, TNC levels were significantly associated with positivity of anti-dsDNA (*p* = 0.03) and anti-nucleosome antibodies (*p* = 0.008). Flares defined by a need to escalate immunosuppressive therapy were captured more frequently and earlier than flares defined by standard activity indices. Higher baseline levels of serum TNC presented a significantly greater risk of flare (i) [hazard ratio (HR) 1.39, 95 % confidence interval (CI) 1.11–1.73] or (ii) (HR 1.25, 95 % CI 1.02–1.52) but not of flares (iii) or (iv). The baseline serum TNC level was the single most important independent predictor of flare (i) compared with conventional biomarkers.

**Conclusions:**

TNC is not disease-specific, but it seems to indicate the activity of SLE and may predict the need to escalate immunosuppressive therapy. TNC levels may thus serve as a useful activity-specific and predictive biomarker in SLE.

**Electronic supplementary material:**

The online version of this article (doi:10.1186/s13075-015-0862-4) contains supplementary material, which is available to authorized users.

## Introduction

Systemic lupus erythematosus (SLE) is a chronic autoimmune disease characterized by a wide range of manifestations involving nearly all organs, including the skin, kidney, lung, brain, heart and joints. Its precise aetiopathogenesis remains unclear. Diverse serological and clinical manifestations as well as unpredictable flares and remissions are observed among patients with SLE, and they present a challenge for the evaluation of disease activity and administration of appropriate treatment. Although clinical assessment is the cornerstone of management of patients with SLE, these evaluations are limited and require additional instruments to confirm the diagnosis and determine disease activity.

Traditional serological biomarkers such as anti-dsDNA antibodies and complement levels have been proven to be neither reliable indicators of disease activity [1, 2] nor predictors of impending disease flares [3]. The lack of useful biomarkers for SLE hampers assessment of disease activity and impedes the evaluation of treatment response. For this reason, there is growing interest in the exploration of new biomarkers for use as surrogate markers of disease activity and/or to predict flares of the disease.

Tenascin-C (TNC) is a large extracellular matrix glycoprotein that belongs to the damage-associated molecular patterns family [4]. Little TNC is found in most healthy adult tissues, because it is specifically induced and tightly controlled during acute inflammation and persistently expressed during chronic inflammation [5–11]. The induction of TNC is highly associated with a wide range of diseases related to inflammation, including pneumonitis [12], hepatitis [13], inflammatory bowel disease [14], myocarditis [15], atherosclerosis [16], obesity [17], rheumatoid arthritis [18] and the enthesitis-related arthritis category of juvenile idiopathic arthritis [19]. An early inflammatory response is generally associated with enhanced TNC levels, both in the plasma and in tissue [6]. Thus, plasma levels of TNC have been shown to be useful indicators for chronic hepatitis C [13], inflammatory bowel disease [14] and myocarditis [15].

To date, no researchers have reported whether circulating TNC levels could reflect disease activity and/or early tissue damage in SLE. Using clinical and laboratory data from our prospective cohort of patients with SLE, we investigated the association of serum TNC levels with Systemic Lupus Erythematosus Disease Activity Index 2000 (SLEDAI-2 K) scores and conventional laboratory markers of disease activity, such as anti–double-stranded DNA antibodies (anti-dsDNA), C3, C4 and anti-nucleosome antibodies. Moreover, we tested the clinical utility of serum TNC levels for the identification of patients with active disease and the prediction of disease flares.

## Material and methods

### Subjects and data collection

Fifty-nine patients fulfilling the revised 1997 American College of Rheumatology classification criteria for SLE [20] were recruited from the Institute of Rheumatology, Prague, and prospectively followed according to a predefined protocol. Clinical and laboratory assessments were performed at baseline, after 3 months, after 6 months and every 6 months thereafter. At each per-protocol visit, disease activity was assessed using the SLEDAI-2 K [21] and the British Isles Lupus Assessment Group disease activity index (BILAG-2004) [22], and the current dosage of glucocorticoids (GC) and each immunosuppressant (IS) medication prescribed for SLE were recorded, in addition to previous changes in medication updated from the source documentation. Patients were followed longitudinally for a mean of 10.8 months [standard deviation (SD) 6.5]. Healthy controls (HC; n = 65) were also recruited. All study participants were ≥18 years of age, and each participant provided written informed consent. The study design and written consent were approved by our institution’s ethics committee.

### Definitions

Because we were interested mainly in global SLE activity, our primary outcome definitions were based on the SLEDAI-2 K. The scores for the clinical items of the SLEDAI-2 K (c-SLEDAI-2 K) were calculated by subtracting the contribution of hypocomplementaemia and anti-dsDNA positivity from the total SLEDAI-2 K score. Active disease was defined as a SLEDAI-2 K score ≥6 [23]. A disease flare was defined as (i) new/increased GC, (ii) new/increased GC or IS, (iii) an increase in the SLEDAI-2 K ≥3 and (iv) BILAG-2004 A or B flare. For analytical purposes, SLEDAI-2 K items describing the involvement of one organ or tissue were consolidated into a single SLEDAI-2 K domain (neuropsychiatric features = seizure, psychosis, organic brain syndrome, cranial nerve disorder, lupus headache, cerebrovascular accident; renal features = haematuria, proteinuria, pyuria, urinary casts; serositis = pleurisy, pericarditis; haematological features = thrombocytopenia, leukopenia).

### Laboratory analysis

Fasting blood samples were collected from all patients during the baseline visit. The samples were immediately centrifuged and stored at −80 °C. Levels of TNC in the serum samples were determined using the human TNC Large (FN III-B) Assay enzyme-linked immunosorbent assay (ELISA) kit from IBL (Fujioka, Japan). The samples were diluted 400-fold. The absorbance was measured using an ELISA reader (SUNRISE; Tecan, Grödig, Austria) using 450 nm as the primary wavelength. The intra- and inter-assay coefficients of variation for this ELISA were 6.4 % and 3.5 %, respectively, at concentrations of 5.43 ng/ml and 6.55 ng/ml. The sensitivity for this kit was 44 pg/ml.

The routine laboratory and immunological measurements needed for the calculation of the SLEDAI-2 K were performed at every visit, and other routine immunological tests [anti-nuclear antibodies (ANA), ANA line immunoassay (LIA) and anti-nucleosome antibodies) were measured at the baseline visit. ANA antibodies were detected by indirect immunofluorescence (Immuno Concepts, Sacramento, CA, USA) and further characterized by the LIA method (IMTEC, Wiesbaden, Germany). Anti-dsDNA antibodies were detected by immunofluorescence (Immuno Concepts); normal was defined as a negative titre. Anti-nucleosome antibodies were measured by ELISA (EUROIMMUN, Lübeck, Germany); normal was defined as 0–24 U/ml. Complement levels were measured using the AU system with reagents (Beckman Coulter, Brea, CA, USA). The reference range for C3 in the serum was 0.9–1.8 g/L, and for C4 it was 0.1–0.4 g/L.

### Statistical analysis

Continuous variables were expressed as the mean ± SD. Categorical data were summarized as absolute frequencies and percentages. We used univariate and multivariable (with adjustments for age and sex) linear regression analyses to assess the association between the baseline serum TNC levels and clinical and laboratory manifestations of SLE. We derived survival curves using the Kaplan-Meier method, and Cox proportional hazards models were used to analyse the association between baseline serum TNC levels and prospectively measured indicators of disease flares. The results were presented as hazard ratio (HR) and 95 % confidence interval (CI). Serum TNC levels were analysed as a continuous variable, with HRs calculated per 100 ng/ml increment of serum TNC. The relationship between serum TNC levels and the risk of endpoint was examined in unadjusted and multivariable adjusted Cox models. The Akaike information criterion (AIC) was used to compare the ability of each immunological marker to predict disease flares, and the differences between predictive models were tested using the likelihood ratio test. Receiver operating characteristic (ROC) curve analysis was performed using data from the inception visit to establish the optimal discriminatory threshold to identify patients with active disease (defined as SLEDAI-2 K ≥ 6) and to predict (i) flares based on TNC levels. A two-tailed *p* value <0.05 was considered statistically significant. Statistical analyses were performed using IBM SPSS version 22 software (IBM SPSS, Armonk, NY, USA).

## Results

### Demographic and clinical characteristics of patients with SLE

The SLE cohort consisted of 93 % women with a mean (±SD) age of 44 ± 16 years. At baseline, 95 % were ANA-positive, 39 % were anti-dsDNA–positive, 46 % were anti-nucleosome antibody–positive and 49 % had low serum complement (C3, C4 or both). The mean (±SD) SLEDAI-2 K was 3.7 ± 3.5, the disease duration was 7 ± 7 years, 33 % had active disease as defined by a SLEDAI-2 K ≥6, 59 % were using oral GC, 42 % were using anti-malarials and 20 % were using IS. The HC were somewhat older, and males were more often represented. The baseline characteristics of the SLE cohort and the demographics of the HC are summarized in Table 1.

**Table 1:** Baseline characteristics

	SLE (n = 59)	Healthy controls (n = 65)
Female sex	55 (93 %)	45 (69 %)
Age, yr	44 ± 16	48 (14)
Caucasian	59 (100 %)	65 (100 %)
Disease duration, yr	7 ± 7	
SLICC/ACR Damage Index	0.8 ± 1.4	
SLEDAI-2 K	3.7 ± 3.5	
cSLEDAI-2 K (only clinical SLEDAI-2 K items)	2.2 ± 3.0	
SLEDAI-2 K ≥4	30 (53 %)	
SLEDAI-2 K ≥6	19 (33 %)	
Any SLEDAI-2 K clinical features	26 (45 %)	
Neuropsychiatric features^a^	2 (3 %)	
Vasculitis^a^	0 (0 %)	
Arthritis^a^	10 (17 %)	
Myositis^a^	0 (0 %)	
Renal features^a^	8 (14 %)	
Rash^a^	9 (15 %)	
Alopecia^a^	5 (9 %)	
Mucosal ulcers^a^	0 (0 %)	
Serositis^a^	1 (2 %)	
Haematological features^a^	3 (5 %)	
Fever^a^	0 (0 %)	
Increased DNA binding^a^	22 (38 %)	
Low complement^a^	28 (48 %)	
Anti-nucleosome antibody–positive	25 (46 %)	
Oral glucocorticoids	35 (59 %)	
Immunosuppressants	15 (25 %)	

*ANA* anti-nuclear antibodies, *SLEDAI-2 K* Systemic Lupus Erythematosus Disease Activity Index 2000, *anti-dsDNA* anti–double-stranded DNA, *SLICC*/ACR Systemic Lupus International Collaborating Clinics/American College of Rheumatology, *SLE* systemic lupus erythematosus

Data are presented as number and percentage or mean and standard deviation

^a^According to SLEDAI-2 K definitions; renal, haematological, serositis and neuropsychiatric SLEDAI-2 K features were merged into one item (see Definitions section in text)

### Serum TNC levels in patients with SLE and healthy controls

**Fig. 1:**
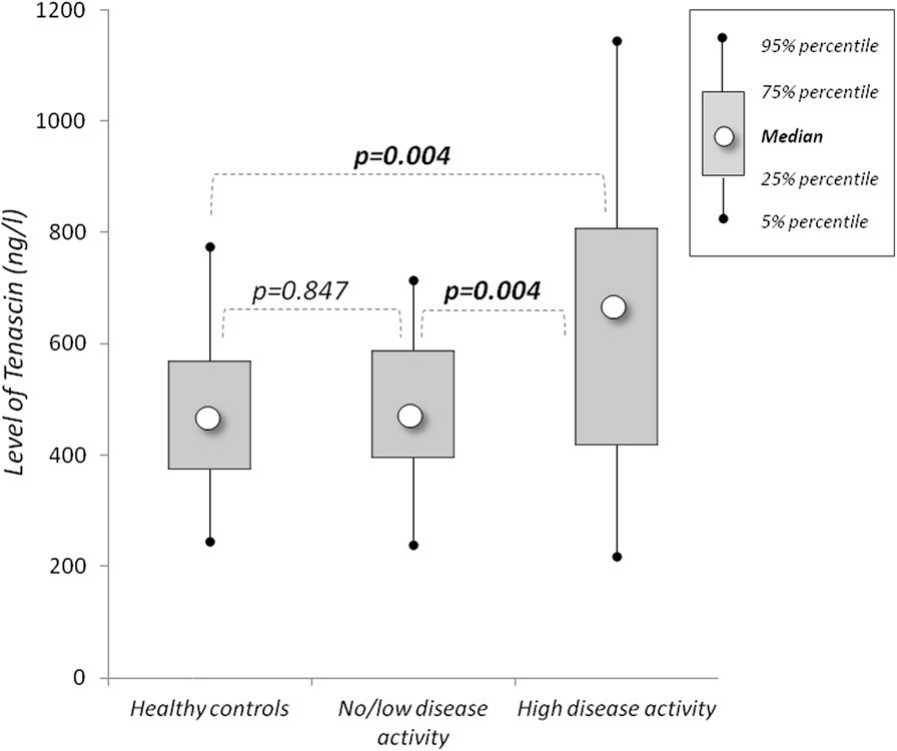
Mean levels of tenascin-C in healthy controls and patients with systemic lupus erythematosus with low [Systemic Lupus Erythematosus Disease Activity Index 2000 (SLEDAI-2 K) <6] and high (SLEDAI-2 K ≥6) disease activity

There was no significant difference in the mean levels of TNC between the patients with SLE and HC (533 ± 192 ng/ml vs. 487 ± 164 ng/ml, *p* = 0.151). However, in patients with SLE with active disease (SLEDAI-2 K ≥6) the TNC levels were significantly higher than in the HC (634 ± 254 ng/ml vs. 487 ± 164 ng/ml, *p* = 0.004) or patients with no or low disease activity (634 ± 254 ng/ml vs. 481 ± 135 ng/ml, *p* = 0.004) (Fig. 1). We found no association between age, sex and TNC levels (see Additional file 1).

### Association between circulating levels of TNC and measures of disease activity

A cross-sectional correlation and univariate linear regression analysis between serum TNC levels and SLEDAI-2 K (β = 14, 95 % CI −1 to 29, *r* = 0.25, *p* = 0.061) and c-SLEDAI-2 K (β = 16, 95 % CI −0.7 to 33, *r* = 0.25, *p* = 0.060) at baseline visit showed a trend towards a positive correlation, although the results were not statistically significant. Patients with SLE with active involvement of at least one SLEDAI-2 K domain (see Definitions section) have significantly higher TNC levels than patients with no clinical involvement according to SLEDAI-2 K (β = 108, 95 % CI 8–207, *p* = 0.035), and patients with any active item in the renal SLEDAI-2 K domain had significantly higher TNC levels than patients without active renal SLEDAI-2 K features (see Table 2 and Additional file 2). The cross-sectional associations between TNC and BILAG-2004 organ domains are shown in Additional file 2. (Note that the renal BILAG-2004 domain could not be properly assessed at inception visit, while its evaluation is heavily dependent on previous measurements which were not captured in our database.)

**Table 2:** Cross-sectional associations between serum TNC levels and clinical and laboratory parameters of patients with SLE at the inception visit (univariate and age- and sex-adjusted regression analyses)

	Univariate analyses		Age– and sex–adjusted analyses	
Variable	β value^b^ (95 % CI)	*p* Value	β value^b^ (95 % CI)	*p* Value
Patients with SLE vs. HC	46 (−17 to 110)	0.151	44 (−24 to 112)	0.205
Patients with SLE with SLEDAI-2 K ≥6 vs. HC	147 (50–245)	**0.004**	139 (34– 245)	**0.010**
Patients with SLE with SLEDAI-2 K ≥6 vs. SLEDAI-2 K <6	153 (50–256)	**0.004**	161 (54–267)	**0.004**
Patients with SLE (categorical variables)				
Any SLEDAI-2 K clinical features (yes vs. no)	107.8 (8.1–207.4)	**0.035**	108.4 (3.6–213.3)	**0.043**
Neuropsychiatric clinical features^a^ (yes vs. no)	−211.9 (−488.9 to 65.2)	0.131	−202.5 (−485.8 to 80.8)	0.158
Vasculitis^a^ (yes vs. no)	–	–	–	–
Arthritis^a^ (yes vs. no)	48.3 (−87.7 to 184.2)	0.480	46.0 (−94.4 to 186.4)	0.514
Myositis^a^ (yes vs. no)	–	–	–	–
Renal features^a^ (yes vs. no)	265.2 (133.5–396.9)	**<0.001**	269.7 (135.9–403.5)	**<0.001**
Rash^a^ (yes vs. no)	−2.1 (−144.6 to 140.4)	0.977	−9.1 (−154.9 to 136.6)	0.901
Alopecia^a^ (yes vs. no)	−84.6 (−267.0 to 97.8)	0.357	−85.0 (−270.7 to 100.7)	0.363
Mucosal ulcers^a^ (yes vs. no)	–	–	–	–
Serositis^a^ (yes vs. no)	231.1 (−160.4 to 622.6)	0.242	216.6 (−187.5 to 620.7)	0.287
Haematological features^a^ (yes vs. no)	20.4 (−212.5 to 253.3)	0.861	9.1 (−231.3 to 249.5)	0.940
Fever^a^ (yes vs. no)	–	–	–	–
Anti-dsDNA antibodies IF (positive vs. negative)	115 (12–218)	**0.029**	112 (3–221)	**0.044**
Complement C3/C4 (low vs. normal)	−4 (−107 to 99)	0.938	−14 (−123 to 94)	0.793
Anti-nucleosome antibodies (positive vs. negative)	138 (38–238)	**0.008**	131 (30–234)	**0.013**
Patients with SLE (continuous variables)				
SLEDAI-2 K	14 (−1 to 29)	0.061	14 (−1.5 to 30)	0.074
cSLEDAI-2 K (only clinical SLEDAI-2 K items)	16 (−1 to 33)	0.060	16 (−1.0 to 34)	0.065
C3, g/L	−9 (−209 to 192)	0.931	5 (−205 to 216)	0.958
C4, g/L	−232 (−695 to 230)	0.319	−210 (−694 to 273)	0.386
Anti-nucleosome antibodies, U	−0.2 (−0.7 to 0.3)	0.403	−0.2 (−0.7 to 0.3)	0.460
Anti-dsDNA antibodies, titre	27 (−22 to 75)	0.266	30 (−21 to 81)	0.230
Urinary protein/creatinine ratio, mg/mmol	270 (101–439)	**0.002**	271 (98–444)	**0.003**

*CI* confidence interval, *HC* healthy controls, *IF* immunofluorescence, *anti-dsDNA* anti–double-stranded DNA, *SLEDAI-2 K* Systemic Lupus Erythematosus Disease Activity Index 2000

^a^According to SLEDAI-2 K definitions; renal, haematological, serositis and neuropsychiatric SLEDAI-2 K features were merged into one item (see Definitions section of text)

^b^The regression coefficient β corresponds to the difference in TNC levels between groups (when assessing categorical variables) or to the change in TNC associated with a 1 unit increase in the assessed variable (when assessing continuous variables)

Boldface type indicates statistically significant values

### Serum TNC levels discriminate between active and inactive disease

**Fig. 2:**
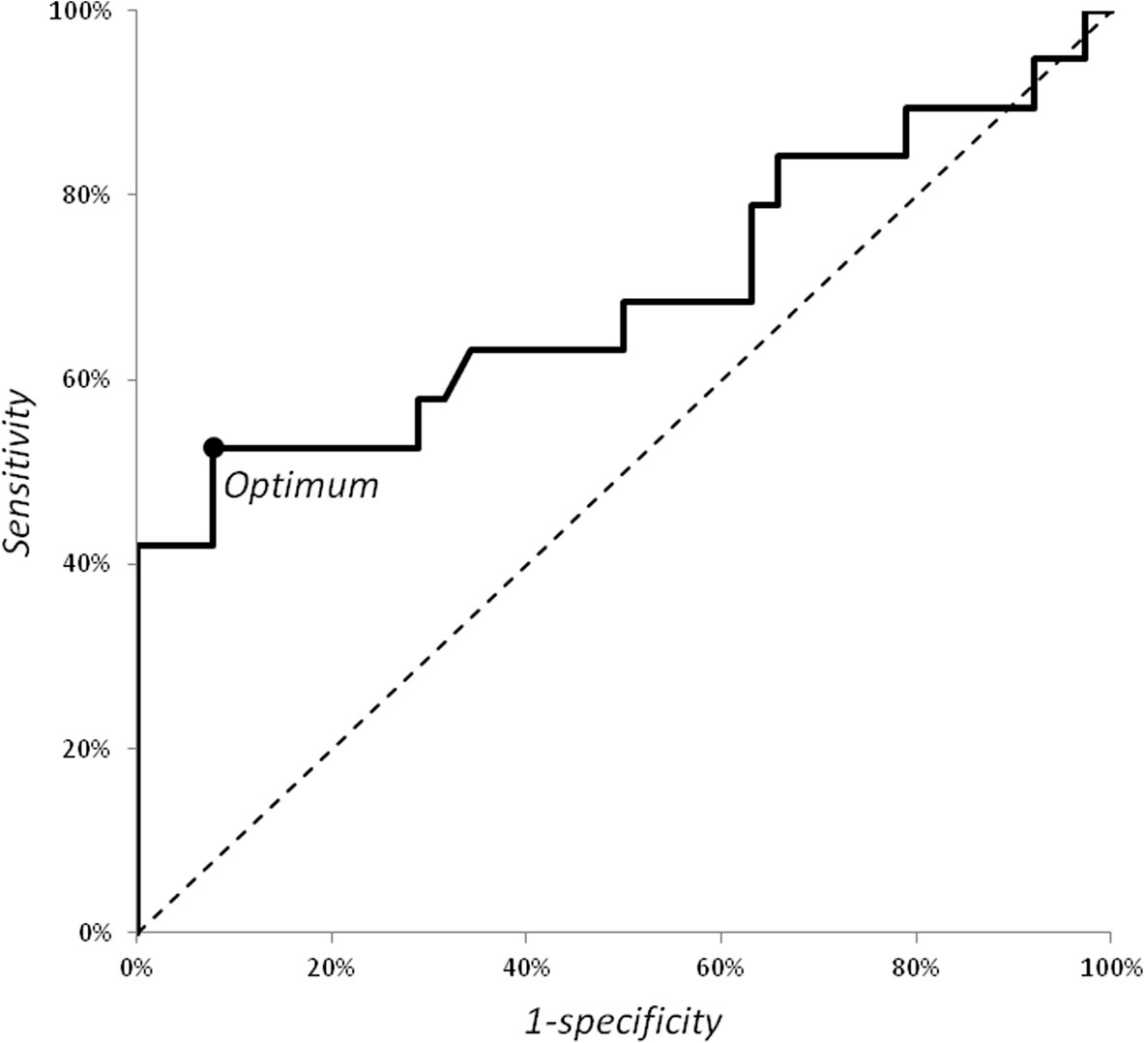
Receiver operating characteristic curve analysis of tenascin (TNC) serum levels as a predictor of active systemic lupus erythematosus (defined as Systemic Lupus Erythematosus Disease Activity Index 2000 ≥ 6). At the optimal cutoff point of 659 ng/ml, the area under the curve for TNC serum levels that discriminated between active and inactive disease was 0.69 (95 % CI 0.53–0.86, *p* = 0.02) with a sensitivity of 53 % and specificity of 92 %

ROC curve analysis was performed to establish the optimal discriminatory threshold to identify patients with active disease (defined as SLEDAI-2 K ≥6) based on TNC levels (Fig. 2). At the optimal cutoff point of 659 ng/ml, the area under the curve for TNC serum levels that discriminated between active and inactive disease was 0.69 (95 % CI 0.53–0.86, *p* = 0.02) with a sensitivity of 53 % and specificity of 92 %.

### Association between circulating levels of TNC and conventional laboratory parameters

A cross-sectional univariate linear regression and/or correlation analysis between serum TNC levels and other laboratory measurements in patients with SLE at the initial visit was performed (Table 2 and Additional file 3a, b). The TNC levels in patients with SLE were positively correlated with positivity for anti-dsDNA antibodies (β = 115, 95 % CI 12–218, *p* = 0.029) and anti-nucleosome antibodies (β = 138, 95 % CI 38–238, *p* = 0.008). Associations between TNC and these parameters were statistically significant even after adjustment for age and sex. In contrast, we did not find a significant association between serum TNC levels and C3/C4 levels.

### Serum TNC levels are predictive of disease flares

**Fig. 3:**
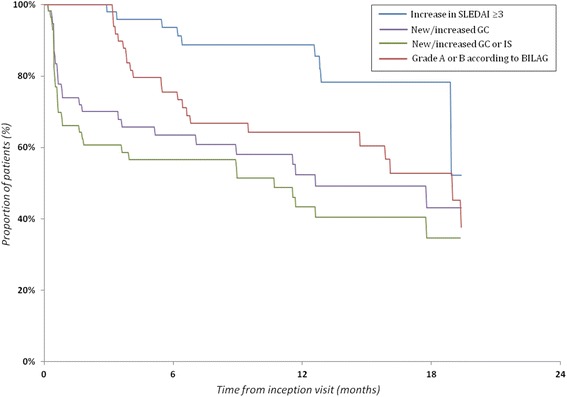
Differential in time to flare according to the definitions of flare used. Systemic Lupus Erythematosus Disease Activity Index 2000 (SLEDAI) and British Isles Lupus Assessment Group disease activity index (BILAG) scores were collected only at prespecified time points (months 0, 3 and 6 and then every 6 months; however, the graphed points are exact dates when the per-protocol visits actually occurred), and reflected only the systemic lupus erythematosus activity within a maximum 30-day time window before each visit. Changes in glucocorticoids and/or immunosuppressive therapy were tracked using the source documentation, and thus these data have finer granularity in time (analysis with the Kaplan-Meier approach for survival estimation). *GC* glucocorticoids, *IS* immunosuppressants

Flares defined by a need to escalate immunosuppressive therapy [(i), (ii)] were captured more frequently and earlier than flares defined by standard activity indices [(iii), (iv)] (Fig. 3). Higher levels of serum TNC presented a significantly greater risk of flare (i) (defined as the need to start or escalate GC) (HR 1.39, 95 % CI 1.11–1.73) or (ii) (defined as the need to start or escalate any IS) (HR 1.25, 95 % CI 1.02–1.52), but not of flare (iii) (defined as an increase in SLEDAI-2 K ≥3) (HR 1.19, 95 % CI 0.87–1.63) or (iv) defined as a BILAG-2004 flare A or B (Table 3).

**Table 3:** Performance of baseline tenascin-C levels to predict disease flares (Cox proportional hazards analysis)

	Univariate analyses		Age and sex adjusted analyses	
Flare definition	HR (95 % CI)	*p* Value	HR (95 % CI)	*p* Value
Tenascin as continuous variable				
(i) New/increased GC	1.39 (1.11–1.73)	**0.004**	1.37 (1.11–1.70)	**0.004**
(ii) New/increased GC or IS	1.25 (1.02–1.52)	**0.028**	1.23 (1.01–1.49)	**0.035**
(iii) Increase in SLEDAI-2 K ≥3	1.19 (0.87–1.63)	0.277	1.21 (0.86–1.68)	0.270
(iv) BILAG-2004 flare A or B	1.10 (0.91–1.34)	0.323	1.09 (0.89–1.32)	0.403
Tenascin as categorical variable (>659 ng/ml)^a^				
(i) New/increased GC	3.77 (1.60–8.88)	**0.002**	3.57 (1.48–8.59)	**0.005**
(ii) New/increased GC or IS	2.45 (1.10–5.46)	**0.028**	2.23 (0.98–5.08)	0.056
(iii) Increase in SLEDAI-2 K ≥3	1.42 (0.28–7.21)	0.672	1.52 (0.27–8.64)	0.636
(iv) BILAG-2004 flare A or B	1.74 (0.75–4.04)	0.197	1.64 (0.70–3.88)	0.257

*BILAG-2004* British Isles Lupus Assessment Group disease activity index, *CI* confidence interval, *HR* hazard ratio, *IS* immunosuppressants, *GC* glucocorticoids, *SLEDAI-2 K* Systemic Lupus Erythematosus Disease Activity Index 2000

^a^The threshold value of 659 ng/ml for tenascin-C (TNC) was generated using receiver operating characteristic curve analysis of the relationship between active systemic lupus erythematosus (SLEDAI-2 K ≥6) and baseline TNC

Boldface type indicates statistically significant values

**Fig. 4:**
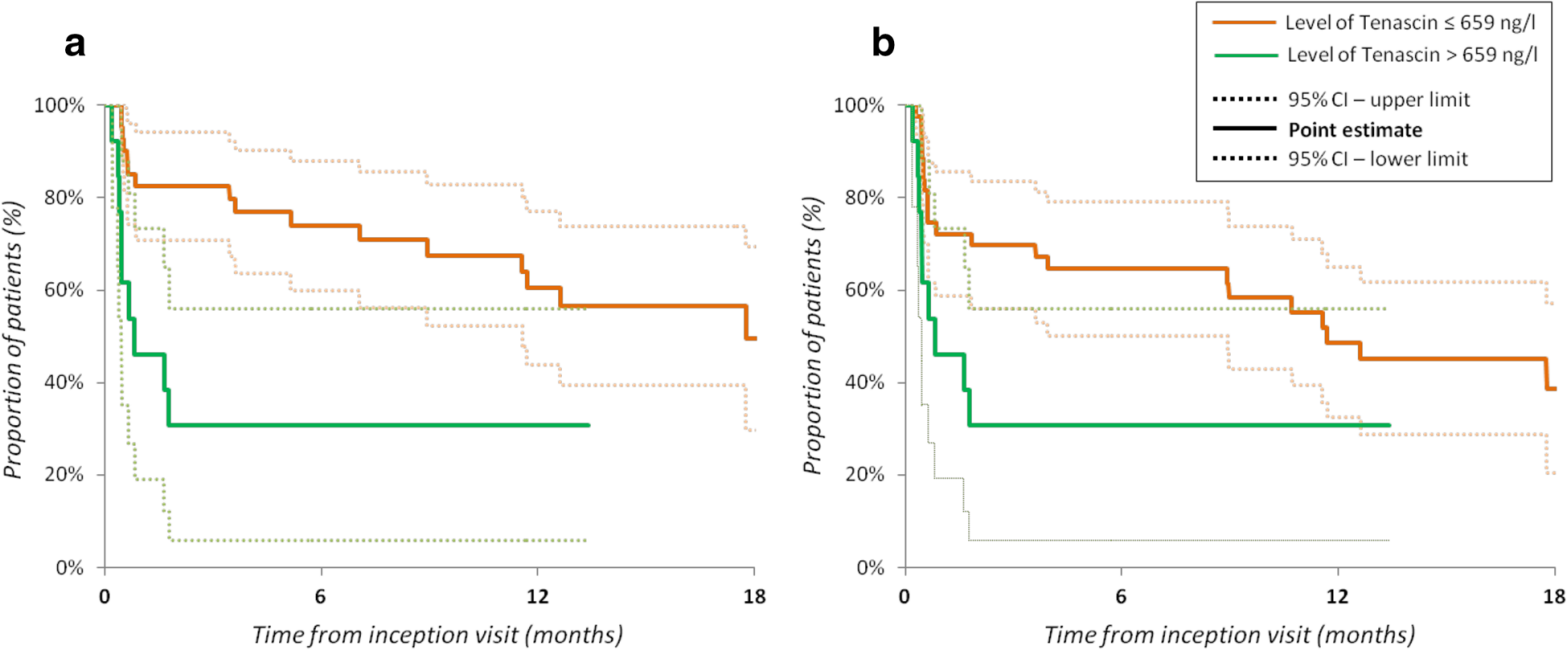
Differentials in time to (**a**) flare (i) and (**b**) flare (ii) according to the baseline level of serum tenascin-C. Flare (i) was defined as the need to start or escalate glucocorticoids, and flare (ii) was defined as the need to start or escalate any immunosuppressant (analysis with the Kaplan-Meier approach for survival estimation). *CI* confidence interval

We also conducted a separate analysis in which serum TNC levels were treated as a categorical variable. We used the value of 659 ng/ml from the previous ROC curve analysis as a cutoff to identify patients with active disease. This value was almost identical to the value of 654 ng/ml generated by a separate ROC curve analysis to find an optimal discriminatory threshold to identify patients predicted to experience flare (i). In accordance with the result above, the risk of flare (i) or (ii) was significantly higher in the group of patients with higher TNC levels (Table 3, Fig. 4).

### Serum TNC levels outperformed traditional biomarkers in the prediction of flare (i)

Next, we compared the performance of univariate models based either on traditional biomarkers (i.e., anti-dsDNA or anti-nucleosome antibodies and C3 or C4) or serum TNC levels to predict flare (i) using the AIC. As shown in Table 4, serum TNC levels outperformed traditional biomarkers when treated as both a continuous variable and a categorical variable.

**Table 4:** A comparison of the performance of conventional biomarkers vs. tenascin-C to predict the escalation of glucocorticoids in patients with SLE

Cox model	Variable					
Continuous	AIC	C3	C4	Anti-dsDNA	Anti-nucleosome	Tenascin
AIC		165.02	163.52	162.67	161.87	160.40
C3	165.02	1.000	0.220	0.125	0.076	**0.032**
C4	163.52	0.220	1.000	0.357	0.199	0.078
Anti-dsDNA	162.67	0.125	0.357	1.000	0.370	0.132
Anti-nucleosome antibodies	161.87	0.076	0.199	0.370	1.000	0.227
Tenascin	160.40	**0.032**	0.078	0.132	0.227	1.000
Categorical		Low C3	Low C4	Anti-dsDNA+	Anti-nucleosome+	Tenascin+
AIC		165.45	164.44	163.21	160.25	160.06
Low C3	165.45	1.000	0.314	0.134	**0.023**	**0.020**
Low C4	164.44	0.314	1.000	0.268	**0.041**	**0.036**
Anti-dsDNA antibodies+	163.21	0.134	0.268	1.000	0.085	0.076
Anti-nucleosome antibodies+	160.25	**0.023**	**0.041**	0.085	1.000	0.667
Tenascin+	160.06	**0.020**	**0.036**	0.076	0.667	1.000

*anti-dsDNA* anti–double-stranded DNA

This table compares univariate Cox regression models to predict the escalation of therapy by glucocorticoids based on the Akaike information criterion (AIC). The lower the value of the AIC, the better the fit of the model. Predictors are treated in the upper part of the table as continuous variables and in the lower part of the table as categorical variables. Paired comparisons of the quality of each model are illustrated by their respective *p* values. The thresholds for conventional biomarkers were reference values, and a cutoff value of 654 ng/ml for tenascin-C was generated using receiver operating characteristic curve analysis to find the optimal discriminatory threshold to identify patients who would require escalation of glucocorticoids. Boldface type indicates statistically significant values

## Discussion

In this study, we found that high TNC levels reflected disease activity and predicted the escalation of GC or other immunosuppressive therapies. To the best of our knowledge, this is the first study to examine in detail the role of TNC in patients with SLE.

An important prerequisite for testing a biomarker in a new clinical setting is its biological plausibility. SLE is a complex autoimmune disease characterized by enhanced autoantibody formation, excessive proinflammatory cytokine production and damage to multiple organ systems. TNC is a proinflammatory extracellular matrix glycoprotein that has been shown to be involved in the regulation of both innate and adaptive immune systems and to control the expression of various cytokines and the recruitment of immune cells to sites of inflammation or injury [7–11]. Little or no TNC is found in most healthy adult tissues, but it is upregulated under pathological conditions accompanying tissue injury and inflammation in many different organs that may be involved in the SLE disease process, including the joints [8, 18], skin [24], kidney [25], lungs [26], heart [27] and central nervous system [28]. Therefore, TNC seems to be an eligible candidate surrogate marker of ongoing tissue damage and may reflect disease activity or an impending flare. Moreover, TNC expression was previously shown to have some applications in disease diagnosis and outcome prognostication in immune-mediated and other inflammatory diseases [7–19, 24–28].

The initial cross-sectional correlation analyses appeared to support the utility of TNC as a putative SLE biomarker. At the inception visit in our SLE cohort, TNC levels were significantly associated with positivity of anti-dsDNA and anti-nucleosome antibodies. Additionally, there was a trend towards a positive correlation with the SLEDAI-2 K and c-SLEDAI-2 K scores. Interestingly, patients with SLE with active renal involvement (mainly with proteinuria) had significantly higher TNC levels than other patients with SLE. This finding is in line with other observations of increased local expression or increased TNC circulating levels in patients with different types of renal disease [29–31].

During the process of validation of a biomarker for the assessment of disease activity, the first external criterion to be correlated with the biomarker may be dichotomous (i.e., active vs. inactive SLE) [32]. To delineate active disease from inactive disease, we chose the robust definition of SLEDAI-2 K ≥6 that has been used in most clinical SLE trials [23]. We found that mean TNC levels in patients with SLE with active disease were significantly higher than those of patients with SLE with no or low disease activity or those of HC. High serum TNC levels showed 53 % sensitivity and 92 % specificity in a ROC curve analysis. Hence, TNC levels could be a useful marker for distinguishing active from inactive SLE.

We also investigated the ability of serum TNC levels to predict disease flares. Higher baseline levels of serum TNC showed a significantly greater risk of a disease flare, defined as the need to start or escalate GC or other immunosuppressive therapy but not a flare defined as a change in the SLEDAI-2 K ≥3 or a BILAG-2004 A or B flare. There is no clear consensus on the best definition of a flare in SLE or on the minimal clinically important change that would be both sensitive and specific [33]. We used four definitions to define disease flares: two based on a change in immunosuppressive therapy [(i) and (ii)] and two based on a change in SLEDAI-2 K or BILAG-2004 [(iii) and (iv)]. The Kaplan-Meier curves in Fig. 3 illustrate the dynamics of the sensitivity of different flare definitions in our cohort study. SLEDAI-2 K and BILAG-2004 scores were collected only at prespecified time points (months 0, 3 and 6 and then every 6 months) and reflected only the SLE activity within a maximum 30-day time window before each visit. At each per protocol visit, however, the study nurse, by using the source documentation, could retrospectively track any changes in immunosuppressive therapy that had happened since the previous visit, and thus these data have finer granularity in time and reflect more comprehensively the course of the disease. Owing to the size of our study sample, we preferred to have a more sensitive tool. Hence, our primary definition of the flares was based on the recorded new start or dose escalation of GC. Because GC are still considered to be the most effective treatment for SLE, the need to start or escalate GC seems to be both a sensitive and a pragmatic outcome measurement of a disease flare. However, the addition of an immunosuppressant may not always mirror an increase in SLE activity; it may also represent a steroid-sparing strategy. Flare definitions based on change in the SLEDAI-2 K also have several limitations. For example, the SLEDAI-2 K does not capture mild degrees of activity in some organ systems, does not have descriptors for several types of activity (e.g., haemolytic anaemia) and cannot capture worsening of activity in an organ system descriptor [33]. Consequently, even lower SLEDAI-2 K cutoffs may have suboptimal sensitivity to capture SLE exacerbations [34, 35], while BILAG-2004 flare definitions may (as in our study) perform somewhat better [36]. Importantly, we observed flares defined by changes in immunosuppressive therapy rather early after the inception visit (i.e., close to the instant of blood sampling for TNC measurement), while relapses defined by SLEDAI-2 K or BILAG-2004 could occur no earlier than at the time of the next per-protocol visit. Hence, our finding of a possible predictive value of TNC may in fact relate mainly to very early flares and/or reflect worsening of baseline activity.

There is a paucity of validated SLE biomarkers that simultaneously reflect disease activity and, more important, forecast impending flares. Currently, anti-dsDNA antibodies and complement levels are the only serological parameters that are routinely used as activity-specific biomarkers in SLE patient care. However, these traditional biomarkers are not always appropriate for clinical monitoring, because high levels of anti-dsDNA or low levels of C3/C4 are persistent in some patients with lupus. Recently, anti-nucleosome antibodies were also suggested as a useful biomarker that may have additional value when evaluated with traditional biomarkers [37].

We compared the value of univariate models based on either conventional biomarkers (i.e., anti-dsDNA or anti-nucleosome antibodies and C3 or C4) or TNC to predict disease flare (i). In our cohort, serum TNC levels outperformed traditional biomarkers when treated as both a continuous and a categorical variable. It is fair to mention that we used reference threshold values of the conventional biomarkers, while the threshold value for TNC was generated by a ROC curve analysis for this very purpose using our SLE cohort. However, in daily clinical practice, conventional biomarkers are always interpreted in the context of their reference values; hence, such a comparison with a new investigational biomarker is sensible. We did not compare the role of conventional biomarkers with TNC to discriminate between active versus inactive disease, because anti-dsDNA and complement contribute to the SLEDAI-2 K-2 K score on which our definition of active disease was based.

Some questions remain. TNC was shown to correlate with the degree of activity in some diseases and clinical scenarios [13–15], while in others it appeared to reflect subsequent tissue remodelling or irreversible damage [18, 24, 26]. TNC is probably neither disease-specific nor pathology-specific, but rather a more universal and ubiquitous marker of ongoing tissue injury, although its potential for monitoring lupus nephritis may deserve closer evaluation. Further study in patients with SLE is required to elucidate whether fluctuations in TNC levels may be used to herald early and reversible changes (i.e., activity) or to signal ongoing fibrosis and progressive and/or irreversible tissue damage. Interestingly, some data indicate that GC may suppress TNC expression [38]. We did not find any correlation with the current dose of GC (data not shown), and we were unable to measure the therapeutic response to GC in our cohort owing to the limited follow-up duration. A much larger study would be required to evaluate the predictive role of TNC for long-term organ damage accrual in SLE.

Our study has several strengths. To the best of our knowledge, this study is the largest to investigate the role of TNC in SLE to date. Our results seem to be consistent both cross-sectionally (TNC levels discriminated active from inactive disease and were correlated with the positivity of traditional biomarkers) and longitudinally (TNC levels were predictive of future escalations of immunosuppressive therapy).

The study has several limitations. It was performed cross-sectionally in a single SLE cohort with a limited number of patients. We did not have sufficient follow-up data to assess the informational value of dynamic changes in TNC levels and their consistency in serial samples. We also used an open rather than predefined time window for a flare to occur during follow-up after TNC sampling because we were not aware of the “right” model to choose based on the (unknown) TNC pathophysiology in SLE.

## Conclusions

We found that circulating levels of TNC aided in the discrimination of patients with SLE with active disease from HC or patients with no or low disease activity. Moreover, high levels of TNC were associated with an increased risk of the need to start or escalate the dose of GC. Further studies with a larger cohort of patients are required to validate the role of TNC as a useful serum biomarker for monitoring disease activity and predicting flares in patients with SLE.

## Additional files

Additional file 1:
**Comparison of tenascin-C levels between males and females, and between female patients and female healthy controls.** (PDF 386 kb) Additional file 2:
**a** **–g** **Sensitivity analyses showing cross-sectional associations between tenascin-C levels and SLE activity in various organ domains according to SLEDAI-2 K and BILAG-2004 indices at inception visit.** (PDF 853 kb) Additional file 3:
**a** **Cross-sectional associations between serum tenascin-C levels and positivity of anti-ds-DNA antibodies at the inception visit (univariate analysis).** **b** Cross-sectional associations between serum tenascin-C levels and positivity of anti-nucleosome antibodies at the inception visit (univariate analysis). (ZIP 46 kb)

## Abbreviations

AIC: Akaike information criterion
ANA: anti-nuclear antibodies
anti-dsDNA: anti–double-stranded DNA antibody
BILAG: British Isles Lupus Assessment Group disease activity index
CI: confidence interval
c-SLEDAI-2 K: Systemic Lupus Erythematosus Disease Activity Index 2000 clinical items
ELISA: enzyme-linked immunosorbent assay
GC: glucocorticoids
HC: healthy controls
HR: hazard ratio
IF: immunofluorescence
IS: immunosuppressants
LIA: line immunoassay
ROC: receiver operating characteristic
SD: standard deviation
SLE: systemic lupus erythematosus
SLEDAI-2 K: Systemic Lupus Erythematosus Disease Activity Index 2000
SLICC/ACR: Systemic Lupus International Collaborating Clinics/American College of Rheumatology
TNC: tenascin-C
