# Supplementary material for: Serum tenascin-C discriminates patients with active SLE from inactive patients and healthy controls and predicts the need to escalate immunosuppressive therapy: a cohort study
Source: Arthritis Res Ther. 2015 Nov 25;17:341. doi: 10.1186/s13075-015-0862-4 (PMC4660660; doi:10.1186/s13075-015-0862-4)
Supplement: Additional file 1: — Comparison of tenascin-C levels between males and females, and between female patients and female healthy controls. (PDF 386 kb) [file 13075_2015_862_MOESM1_ESM.pdf]

Additional file 1

Comparison of Tenascin-C levels between males and females, and between female patients and female HCs.

	Univariate analyses		Age adjusted analyses		Valid N
**Variable**	**β* (95% CI)**	**p value**	**β* (95% CI)**	**p value**	
All females vs. all males	17.3 (-64.2; 98.9)	0.675	18.5 (-63.3; 100.4)	0.655	122
Female patients vs. female HCs	41.3 (-31.6; 114.3)	0.263	35.4 (-38.6; 109.3)	0.345	100
Female HCs vs. male HCs	9.6 (-79.4; 98.5)	0.831	8.4 (-81.7; 98.5)	0.852	65

** The regression coefficient β corresponds to the difference in TNC levels between groups. HC – healthy controls*
