# Supplementary material for: Serum tenascin-C discriminates patients with active SLE from inactive patients and healthy controls and predicts the need to escalate immunosuppressive therapy: a cohort study
Source: Arthritis Res Ther. 2015 Nov 25;17:341. doi: 10.1186/s13075-015-0862-4 (PMC4660660; doi:10.1186/s13075-015-0862-4)
Supplement: Additional file 2: — a –g Sensitivity analyses showing cross-sectional associations between tenascin-C levels and SLE activity in various organ domains according to SLEDAI-2 K and BILAG-2004 indices at inception visit. (PDF 853 kb) [file 13075_2015_862_MOESM2_ESM.pdf]

Additional files 2a-g

Sensitivity analyses showing cross-sectional associations between Tenascin-C levels and SLE activity in various organ domains according to SLEDAI-2K and BILAG-2004 indices at inception visit.

SLEDAI-2K

Additional file 2a Association of serum levels of Tenascin with the presence of clinical features according to the SLEDAI. Reference category are all patients without involvement in the respective organ domain.

Parameter	Univariate analyses		Age and sex adjusted analyses		*N of patients*
	β** (95% IS)	p-value	β** (95% IS)	p-value	
**Any clinical features (Yes vs. No)**	**107.8 (8.1; 207.4)**	**0.035**	**108.4 (3.6; 213.3)**	**0.043**	*26 vs.32*
Neuropsychiatric clinical features* (Yes vs. No)	-211.9 (-488.9; 65.2)	0.131	-202.5 (-485.8; 80.8)	0.158	*2 vs. 56*
Vasculitis* (Yes vs. No)	-	-	-	-	*0 vs. 58*
Arthritis* (Yes vs. No)	48.3 (-87.7; 184.2)	0.480	46.0 (-94.4; 186.4)	0.514	*10 vs. 48*
Myositis* (Yes vs. No)	-	-	-	-	*0 vs.58*
Renal features * (Yes vs. No)	**265.2 (133.5; 396.9)**	**< 0.001**	**269.7 (135.9; 403.5)**	**< 0.001**	*8 vs.50*
Rash* (Yes vs. No)	-2.1 (-144.6; 140.4)	0.977	-9.1 (-154.9; 136.6)	0.901	*9 vs.49*
Alopecia * (Yes vs. No)	-84.6 (-267.0; 97.8)	0.357	-85.0 (-270.7; 100.7)	0.363	*5 vs.53*
Mucosal ulcers * (Yes vs. No)	-	-	-	-	*0 vs.58*
Serositis* (Yes vs. No)	231.1 (-160.4; 622.6)	0.242	216.6 (-187.5; 620.7)	0.287	*1 vs.57*
Hematological features * (Yes vs. No)	20.4 (-212.5; 253.3)	0.861	9.1 (-231.3; 249.5)	0.940	*3 vs.55*
Fever* (Yes vs. No)	-	-	-	-	*0 vs.58*

** according to SLEDAI definitions (renal, hematological, serositis and neuropsychiatric SLEDAI features were merged into one item - see definitions). **The regression coefficient β corresponds to the difference in TNC levels between groups (when assessing categorical variables) or to the change in TNC associated with a 1 unit increase in the assessed variable (when assessing continuous variables). (N=58, 1 patient had incomplete SLEDAI questionnaire).*

Additional file 2b Association of serum levels of Tenascin with the presence of clinical features acc. to the SLEDAI. Reference category are all patients with clinical SLEDAI=0.

**Parameter**	**Univariate analyses**		**Age and sex adjusted analyses**		***N of patients***
	**β** (95% IS)**	**p-value**	**β** (95% IS)**	**p-value**	
No clinical features acc. to SLEDAI	*reference category*		*reference category*		*32*
**Any clinical features**	**107.8 (8.1; 207.4)**	**0.035**	**108.4 (3.6; 213.3)**	**0.043**	*26 vs. 32*
Neuropsychiatric clinical features*	-156.3 (-356.4; 43.8)	0.122	-146.3 (-344.6; 52.0)	0.142	*2 vs. 32*
Vasculitis	-	-	-	-	*0 vs. 32*
Arthritis	88.3 (-16.7; 193.2)	0.097	104.5 (-3.3; 212.3)	0.057	*10 vs. 32*
Myositis	-	-	-	-	*0 vs. 32*
Renal features *	**276.9 (157.5; 396.4)**	**< 0.001**	**289.1 (168.2; 409.9)**	**< 0.001**	*8 vs. 32*
Rash	46.6 (-73.7; 166.8)	0.438	40.9 (-83.6; 165.5)	0.510	*9 vs. 32*
Alopecia	-29.0 (-164.0; 106.0)	0.665	-12.8 (-147.8; 122.2)	0.848	*5 vs. 32*
Mucosal ulcers	-	-	-	-	*0 vs. 32*
Serositis	275.4 (-2.2; 553.0)	0.052	**298.6 (15.2; 582.0)**	**0.040**	*1 vs. 32*
Hematological features *	67.7 (-96.4; 231.8)	0.407	90.7 (-77.5; 259.0)	0.280	*3 vs. 32*
Fever	-	-	-	-	*0 vs. 32*

** according to SLEDAI definitions (renal, hematological, serositis and neuropsychiatric SLEDAI features were merged into one item - see definitions). **The regression coefficient β corresponds to the difference in TNC levels between groups (when assessing categorical variables) or to the change in TNC associated with a 1 unit increase in the assessed variable (when assessing continuous variables). (N=58, 1 patient had incomplete SLEDAI questionnaire).*

Additional file 2c Association of serum levels of Tenascin with the presence of clinical features acc. to the SLEDAI . Reference category are all patients with SLEDAI=0.

**Parameter**	**Univariate analyses**		**Age and sex adjusted analyses**		***N of patients***
	**β** (95% IS)**	**p-value**	**β** (95% IS)**	**p-value**	
No SLEDAI symptoms	*reference category*		*reference category*		*17*
Any clinical features	108,7 (-16,8; 234,3)	0,088	105,8 (-30,1; 241,8)	0,123	*26 vs. 17*
Neuropsychiatric clinical features*	-155.3 (-335.3; 24.7)	0.086	-156.2 (-344.6; 32.2)	0.098	*2 vs. 17*
Vasculitis	-	-	-	-	*0 vs. 17*
Arthritis	89.2 (-22.3; 200.7)	0.112	102.0 (-20.5; 224.5)	0.098	*10 vs. 17*
Myositis	-	-	-	-	*0 vs. 17*
**Renal**	**277.9 (148.9; 406.9)**	**< 0.001**	**281.1 (142.1; 420.1)**	**< 0.001**	*8 vs. 17*
Rash	47.5 (-87.9; 182.9)	0.476	43.4 (-106.4; 193.2)	0.554	*9 vs. 17*
Alopecia	-28.1 (-160.0; 103.8)	0.662	-13.5 (-154.0; 127.0)	0.843	*5 vs. 17*
Mucosal ulcers	-	-	-	-	*0 vs. 17*
**Serositis**	**276.4 (34.3; 518.4)**	**0.028**	**312.7 (38.0; 587.4)**	**0.029**	*1 vs. 17*
Hematological	68.6 (-79.8; 217.0)	0.344	101.1 (-66.1; 268.3)	0.218	*3 vs. 17*
Fever	-	-	-	-	*0 vs. 17*

** according to SLEDAI definitions (renal, hematological, serositis and neuropsychiatric SLEDAI features were merged into one item - see definitions). **The regression coefficient β corresponds to the difference in TNC levels between groups (when assessing categorical variables) or to the change in TNC associated with a 1 unit increase in the assessed variable (when assessing continuous variables). (N=58, 1 patient had incomplete SLEDAI questionnaire).*

BILAG 2004 (note that the renal BILAG domain could not be properly assessed at inception visit, while its evaluation is heavily dependent on previous measurements which were not captured in our database).

Additional file 2d Association of serum levels of Tenascin with the presence of **grade A or B acc. to BILAG** in BILAG domains (N=59). Reference category are all patients without involvement in the respective organ domain.

**Domain of BILAG:**	**Univariate analyses**		**Age and sex adjusted analyses**		***Number of patients***
	**β* (95% CI)**	**p-value**	**β* (95% CI)**	**p-value**	
Any of BILAG domain (Y vs. N)	-8.6 (-127.7; 110.6)	0.886	-15.8 (-138.2; 106.6)	0.797	*14 vs. 45*
Constitutional (Y vs. N)	121.4 (-270.1; 512.9)	0.537	128.0 (-269.1; 525.2)	0.521	*1 vs.58*
Mucocutaneous (Y vs. N)	-0.9 (-182.9; 181.2)	0.992	-15.5 (-204.7; 173.6)	0.870	*5 vs.54*
Neuropsychiatric (Y vs. N)	-204.3 (-428.6; 20.1)	0.074	-201.8 (-429.8; 26.2)	0.082	*3 vs.56*
Musculoskeletal (Y vs. N)	-14.6 (-294.8; 265.5)	0.917	-19.9 (-305.1; 265.3)	0.889	*2 vs.57*
Cardiorespiratory (Y vs. N)	231.7 (-156.3; 619.7)	0.237	217.3 (-183.0; 617.5)	0.281	*1 vs.58*
Gastrointestinal (Y vs. N)	-	-	-	-	*0 vs.59*
Ophthalmic (Y vs. N)	-	-	-	-	*0 vs.59*
Renal (Y vs. N)	N/A	N/A	N/A	N/A	N/A
Hematological (Y vs. N)	-5.5 (-398.3; 387.3)	0.978	-14.8 (-414.6; 385.0)	0.941	*1 vs.58*

** The regression coefficient β corresponds to the difference in TNC levels between groups (when assessing categorical variables) or to the change in TNC associated with a 1 unit increase in the assessed variable (when assessing continuous variables). N/A – not applicable*

Additional file 2e Association of serum levels of Tenascin with the presence of **grade A, B or C acc. to BILAG** in BILAG domains (N=59). Reference category are all patients without involvement in the respective organ domain.

**Domain of BILAG:**	**Univariate analyses**		**Age and sex adjusted analyses**		***Number of patients***
	**β* (95% CI)**	**p-value**	**β* (95% CI)**	**p-value**	
Any of BILAG domain (Y vs. N)	82.6 (-34.6; 199.7)	0.164	78.2 (-42.9; 199.2)	0.201	*45 vs. 14*
Constitutional (Y vs. N)	-20.5 (-300.7; 259.6)	0.884	-13.4 (-298.2; 271.4)	0.925	*2 vs. 57*
Mucocutaneous (Y vs. N)	10.8 (-111.5; 133.1)	0.860	8.8 (-115.4; 132.9)	0.888	*13 vs. 46*
Neuropsychiatric (Y vs. N)	-204.3 (-428.6; 20.1)	0.074	-201.8 (-429.8; 26.2)	0.082	*3 vs. 56*
Musculoskeletal (Y vs. N)	78.7 (-49.8; 207.2)	0.225	80.4 (-50.1; 210.8)	0.222	*11 vs. 48*
**Cardiorespiratory (Y vs. N)**	**249.2 (28.1; 470.4)**	**0.028**	**252.0 (27.7; 476.4)**	**0.028**	*3 vs. 56*
Gastrointestinal (Y vs. N)	-	-	-	-	*0 vs. 59*
Ophthalmic (Y vs. N)	-	-	-	-	*0 vs. 59*
Renal (Y vs. N)	N/A	N/A	N/A	N/A	N/A
Hematological (Y vs. N)	95.4 (-5.4; 196.3)	0.063	92.8 (-14.7; 200.3)	0.089	*36 vs. 23*

** The regression coefficient β corresponds to the difference in TNC levels between groups (when assessing categorical variables) or to the change in TNC associated with a 1 unit increase in the assessed variable (when assessing continuous variables). N/A – not applicable*

Additional file 2f Association of serum levels of Tenascin with the presence of **grade A or B acc. to BILAG** in each BILAG domain. Reference category are all patients with BILAG=D or E in all BILAG domains. (N=59).

**Domain of BILAG:**	**Univariate analyses**		**Age and sex adjusted analyses**		***Number of patients***
	**β* (95% CI)**	**p-value**	**β* (95% CI)**	**p-value**	
No grade A or B in all BILAG domains	*reference category*		*reference category*		*45*
Any of BILAG domain	-8.6 (-127.7; 110.6)	0.886	-15.8 (-138.2; 106.6)	0.797	*14 vs. 45*
Constitutional	117.3 (-241.1; 475.8)	0.513	125.2 (-238.4; 488.8)	0.491	*1 vs. 45*
Mucocutaneous	-2.8 (-180.3; 174.7)	0.974	-20.8 (-205.2; 163.6)	0.821	*5 vs. 45*
Neuropsychiatric	-195.9 (-404.3; 12.4)	0.065	-190.6 (-402.2; 20.9)	0.076	*3 vs. 45*
Musculoskeletal	-16.2 (-273.2; 240.9)	0.900	-19.7 (-281.0; 241.6)	0.880	*2 vs. 45*
Cardiorespiratory	225.8 (-132.7; 584.2)	0.211	210.2 (-160.0; 580.5)	0.258	*1 vs. 45*
Gastrointestinal	-	-	-	-	*0 vs. 45*
Ophthalmic	-	-	-	-	*0 vs. 45*
Renal	N/A	N/A	N/A	N/A	N/A
Hematological	-7.5 (-365.9; 351.0)	0.967	-12.7 (-378.0; 352.6)	0.944	*1 vs. 45*

** The regression coefficient β corresponds to the difference in TNC levels between groups (when assessing categorical variables) or to the change in TNC associated with a 1 unit increase in the assessed variable (when assessing continuous variables). N/A – not applicable*

Additional file 2g Association of serum levels of Tenascin with the presence of **grade A, B or C acc. to BILAG** in each BILAG domain. Reference category are all patients with BILAG=D or E in all BILAG domains. (N=59).

**Domain of BILAG:**	**Univariate analyses**		**Age and sex adjusted analyses**		***Number of patients***
	**β* (95% CI)**	**p-value**	**β* (95% CI)**	**p-value**	
No grade A, B or C in all BILAG domains	*reference category*		*reference category*		*14*
Any of BILAG domain	82.6 (-34.6; 199.7)	0.164	78.2 (-42.9; 199.2)	0.201	*45 vs. 14*
Constitutional	43.1 (-219.4; 305.7)	0.730	41.2 (-231.4; 313.8)	0.749	*2 vs. 14*
Mucocutaneous	71.4 (-72.4; 215.2)	0.316	77.0 (-74.6; 228.5)	0.304	*13 vs. 14*
Neuropsychiatric	-130.9 (-339.0; 77.2)	0.200	-128.1 (-342.8; 86.6)	0.222	*3 vs. 14*
Musculoskeletal	127.0 (-21.2; 275.2)	0.089	141.3 (-16.4; 299.0)	0.076	*11 vs. 14*
**Cardiorespiratory**	**299.6 (78.8; 520.4)**	**0.011**	**304.4 (76.0; 532.8)**	**0.013**	*3 vs. 14*
Gastrointestinal	-	-	-	-	*0 vs. 14*
Ophthalmic	-	-	-	-	*0 vs. 14*
Renal	N/A	N/A	N/A	N/A	N/A
Hematological	100.2 (-14.0; 214.4)	0.084	100.0 (-20.3; 220.2)	0.101	*36 vs. 14*

** The regression coefficient β corresponds to the difference in TNC levels between groups (when assessing categorical variables) or to the change in TNC associated with a 1 unit increase in the assessed variable (when assessing continuous variables). N/A – not applicable*
