# Supplementary figures and images for: Serum tenascin-C discriminates patients with active SLE from inactive patients and healthy controls and predicts the need to escalate immunosuppressive therapy: a cohort study
Source: Arthritis Res Ther. 2015 Nov 25;17:341. doi: 10.1186/s13075-015-0862-4 (PMC4660660; doi:10.1186/s13075-015-0862-4)

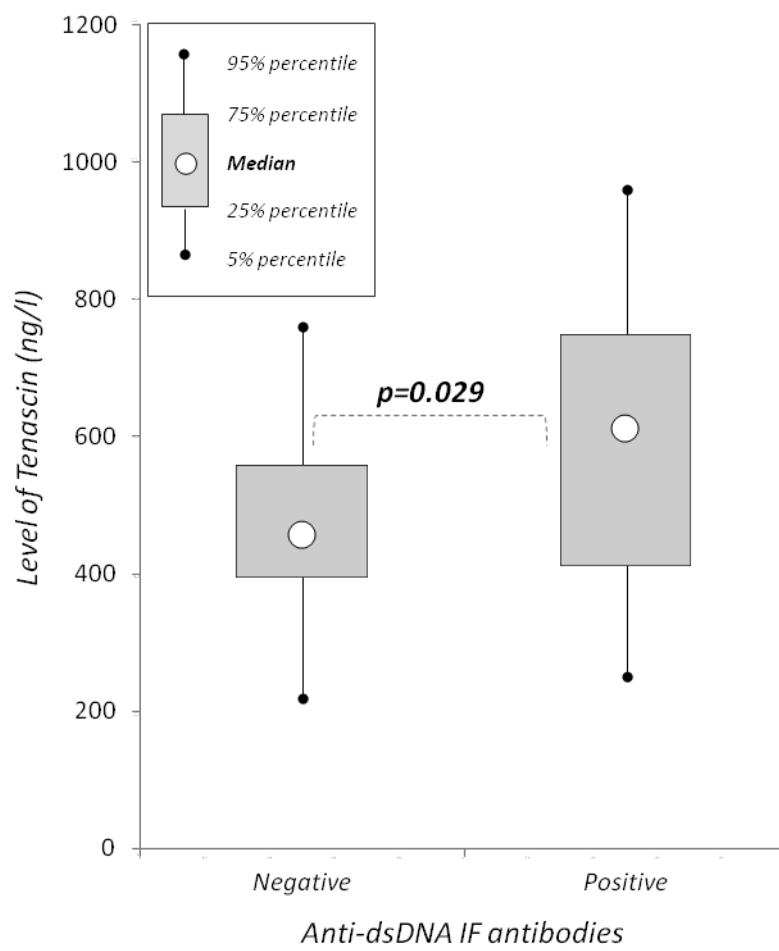

Supplement: Additional file 3: — a Cross-sectional associations between serum tenascin-C levels and positivity of anti-ds-DNA antibodies at the inception visit (univariate analysis). b Cross-sectional associations between serum tenascin-C levels and positivity of anti-nucleosome antibodies at the inception visit (univariate analysis). (ZIP 46 kb) [file 13075_2015_862_MOESM3_ESM.zip › 13075_2015_862_MOESM3a_ESM.pdf]

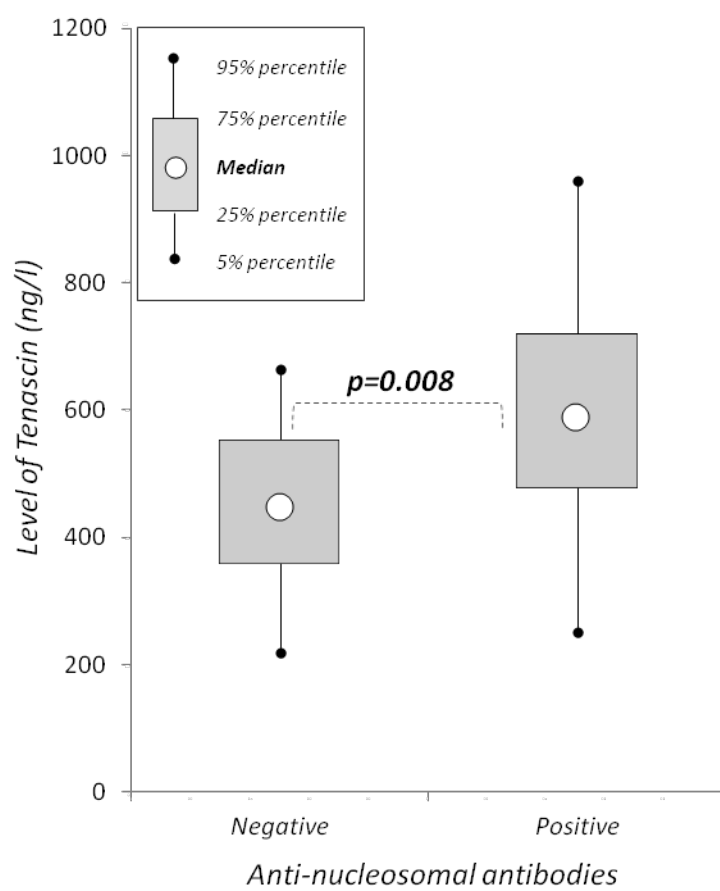

Supplement: Additional file 3: — a Cross-sectional associations between serum tenascin-C levels and positivity of anti-ds-DNA antibodies at the inception visit (univariate analysis). b Cross-sectional associations between serum tenascin-C levels and positivity of anti-nucleosome antibodies at the inception visit (univariate analysis). (ZIP 46 kb) [file 13075_2015_862_MOESM3_ESM.zip › 13075_2015_862_MOESM3b_ESM.pdf]
